# A moral dilemma argument against clinical trials of incentives for kidney donation

**DOI:** 10.1186/s13737-015-0025-9

**Published:** 2015-07-22

**Authors:** G. V. Ramesh Prasad

**Affiliations:** Division of Nephrology, St. Michael’s Hospital, University of Toronto, 61 Queen Street East, 9th Floor, Toronto, ON M5C 2T2 Canada

**Keywords:** Autonomy, Beneficence, Compensation, Dilemma, Organ shortage, Poverty, Transplantation

## Abstract

Commercial transplant tourism results in significant harm to both kidney donors and recipients. However, proponents of incentives for kidney donation assert that proper oversight of the process prevents these harms and also that transplant numbers can be safely increased so that the moral burden of poor end-stage kidney disease outcomes can be alleviated. In a moral dilemma analysis, the principle of preventing donor harm can be dissociated from the principles of providing benefits to the recipient and to society. It is plausible that an incentivized donor is fundamentally different from an uncompensated donor. Incentivized donors can experience harms unrelated to lack of regulation because their characteristics are determined by the incentive superimposed upon a poverty circumstance. Moreover, creating a system of incentivized donation without established national registries for capturing all long-term donor outcomes would be morally inconsistent, since without prior demonstration that donor outcomes are not income or wealth-dependent, a population of incentivized donors cannot be morally created in a clinical trial. Socioeconomic factors adversely affect outcome in other surgical populations, and interventions on income or wealth in these populations have not been studied. Coercion will be increased in families not affected by kidney disease, where knowledge of a new income source and not of a potential recipient is the incentive. In the case of elective surgery such as kidney donation, donor non-maleficence trumps donor autonomy, recipient beneficence, and beneficence to society when there is a conflict among these principles. Yet, we are still faced with the total moral burden of end-stage kidney disease, which belongs to the society that cannot provide enough donor kidneys. Acting according to one arm of the dilemma to prevent donor harm does not erase obligations towards the other, to provide recipient benefit. To resolve the moral burden, as moral agents, we must rearrange our institutions by increasing available donor organs from other sources. The shortage of donor kidneys creates a moral burden for society, but incentives for donation will only increase the total moral burden of end-stage kidney disease.

## Commentary

Transplant tourism is widely condemned [1], but proposals exist for domestic regulated systems of incentives for living kidney donation [2]. Premises such as regulated fair price, third party payment and recipient selection, assurance of long-term health care for donors, and respect for donor and recipient autonomy are commonly employed in advancing arguments for such regulated systems of incentives. Motivation includes increasing total transplant numbers and cost savings to health care systems. Persuasive arguments against incentive-based systems must show them to be morally distinguishable from uncompensated donation [2]. This commentary argues that this moral distinction can be made and that a clinical trial of incentivized donation [2] will therefore lack moral grounding.

## The moral dilemma approach

The moral dilemma is a useful tool that can be used to analyze many life situations. In a moral dilemma, one ought to do x and ought to do y, but one cannot do both simultaneously. When framed as a moral dilemma, on the one hand, we ought to safely increase total kidney transplant numbers, and on the other, we ought to prevent harm to incentivized kidney donors, but we cannot do both together. When properly framed in this manner, each dilemma arm must first be supported, and a resolution then proposed. There is no controversy about safely increasing total transplant numbers, which is the dilemma’s first arm. More contentious is the dilemma’s second arm, according to which regulated incentive systems cause donor harm, and which will therefore be discussed at length. Although poor outcomes in incentivized donors have been reported worldwide [3–5], proponents of incentives indicate that effective regulation will eliminate organ trafficking and poor outcomes; moreover, prohibition of compensation eliminates a chance at poverty alleviation [2].

There are three parties involved in incentivized donation: donors, recipients, and society. In a moral analysis, the principle of preventing donor harm must be dissociated from the principle of providing benefits to recipients or to society. High-quality studies demonstrate safety in regulated uncompensated donation [6]. Uncompensated donors assume the moral burden of their decision to undergo surgery and experience its consequences, all for the recipient’s benefit. Incentivized donor decisions instead are driven by the incentive and are unlinked to the recipient’s benefit. The moral burden for the decision to proceed with surgery rests therefore with the inducing society and to health professionals as society’s representative, but not the donor. Health professionals will not propose pilot studies of a non-clinically indicated cholecystectomy or appendectomy because no other party’s interest is involved, and they will not assume that moral burden. Surveys of patients with kidney disease and the general public about compensated donation [7–10] are used to strengthen arguments for incentives as moral justification, but these surveys are designed to capture the opinion of recipients and society, not that of incentivized donors themselves. Incentivized donor opinion is needed for the moral burden of end-stage kidney disease to be comprehensively assessed.

Incentivized donors may be fundamentally different from other donors and therefore prone to different outcomes. Survey data from previously incentivized donors [3] remains relevant because it is the incentivized donor who is exposed to potential harm. For example, Indian donors experienced health deterioration despite excellent baseline health, and also worsened financially, recommending that others not similarly give up their kidneys [3]. Persistent backache and nephrectomy site pain are phenomena not readily explainable by nephrectomy. In Iran, quality of life scores were inferior to that of the general population [4]. In Pakistan, general health status deteriorated in 98 % of donors deliberately sought out post-surgery [5]. Medical care in these countries cannot readily be dismissed as primitive. It is therefore plausible that adverse outcomes were due not to improper regulation but rather inherent donor characteristics. Poor psychological conditioning resulting from the need to overcome fear-based stress responses instead of experiencing altruistic feelings of reward, or a fundamentally altered self-image post-nephrectomy rather than unrecognized contraindications to nephrectomy or poor surgical technique cannot be prevented by regulation, because a system immune from abuse cannot change donor characteristics regardless of the country of origin of the donors. These donor characteristics may have been selected out by the incentive that has been superimposed upon a poverty circumstance.

Incentivized donors are likely to be in poverty, even in regulated systems. Society does not object to impoverished individuals autonomously assuming risky jobs [2]. However, work options in poverty are often limited to physical labor and could become limited further if existing incentivized donor experiences [3–5] are respected. Employment options in poverty in developing countries are unlikely to differ substantially from those in richer jurisdictions where incentives are being proposed. Poverty may be perpetuated or worsened. Incentives will not improve educational opportunities as a means of poverty alleviation since access to education depends on much more than financial means.

Donors from retrospective long-term donor follow-up studies [6] do not represent the demographic of incentivized donors. Studies of donor outcomes do not focus on economic disadvantage as a variable. Incentivized donation without established national donor registries to capture all donor outcomes is morally inconsistent, since without prior demonstration that donor outcomes are not income or wealth-dependent, a population of incentivized donors cannot morally be created. Up to 25 % of current living donors have medical conditions associated with future health risk [11]. True informed consent is not possible in trials of incentives because there are no prior favorable donor data from non-trial studies of incentives in order to promote clinical equipoise, while true informed consent is possible in the case of uncompensated donation because abundant safety data exists.

With sparse long-term data for incentivized donors, examining the relationship between income and outcomes in other surgical populations is immediately helpful. Socioeconomic factors adversely affect outcome in kidney [12], liver [13–15], and lung [16] transplant recipients. Similar information is available for patients undergoing cardiac surgery [17, 18], prostatectomy [19], neurosurgery [20], and colorectal surgery [21]. Although these findings may not be universal, data from other surgical populations in developed countries needs close examination first, because the underlying mechanism for these poor outcomes remains largely unknown. Even if donation is always cautiously approached in economically disadvantaged candidates, excluding them from incentivized donation would be paradoxical since a purported benefit of incentivized donation is poverty alleviation in donors. Health professionals do not suggest a clinical trial to examine the effects of financial gifts in any other surgical population because no other party’s interest is involved. Incentives to alter adverse outcomes need to be tested first in obligated surgical populations, not to gain advantage from elective surgical populations. Clinical equipoise to justify a clinical trial that intentionally creates a new elective surgical population of incentivized donors is lacking.

Significant financial transactions can occur in all donor-recipient relationships. Even without a reliable method for their capture, transplantation must continue. However, rather than being occasional instances, such transactions are institutionalized by incentivized donation and can only encourage their occurrence in all living donation. Some current living donation also possibly occurs by coercion. A medically suitable family member may be compelled to donate a kidney to a relative. Transplant programs attempt to identify such cases and prevent these transplants. With incentivized donation, a new form of coercion is being introduced, within families that are not affected by kidney disease. Knowledge of a new income source is now the cause of coercion, and so, there will be a new population of coerced donors. Transplant programs support reluctant donors to opt-out of surgery in support of the donor’s autonomy and to prevent long-term, non-surgical harm to the donor. In incentive-based systems, there will be opt-in reasons unrelated to poverty alleviation, some of which will be possibly illegal. Yet, moral consistency would require that the principle of autonomy preservation respect these reasons to donate.

To summarize, the second arm of the moral dilemma, that we ought to prevent harm to incentivized kidney donors, is clearly supported by the lack of favorable outcomes in previously incentivized donors to promote equipoise, no clear relation of these outcomes to deficient regulation, lack of registries to capture all long-term donor outcomes, potential for increased poverty, relationship of wealth or income to outcome in other surgical populations, and increased coercion. Since incentives appear to strengthen and not resolve the moral dilemma, the moral burden from the shortage of available kidneys is increased, not decreased by a regulated system of incentivized donation.

## Resolution of the moral dilemma

Since we cannot both increase total transplant numbers and prevent incentivized donor harm, how do we proceed? One method is specification. Specification works with norms that are not universal generalizations and allow us to articulate our reasons openly and publicly [22]. Specification narrows a norm by adding more information, preventing vagueness contained in terms like “fair compensation” or “dignity and appreciation.” For incentivized donation, additional specifications that border on impossible to fulfil are required. Favorable data from existing incentive systems, study of baseline wealth on outcomes from kidney donor registries, and study of altering wealth by payment on outcomes in obligated, non-elective surgical populations are required to meet the basic moral norm for a clinical trial of incentives for donation, however, limited in scope.

Beauchamp and Childress describe autonomy, beneficence, non-maleficence, and justice as the normative standards of conduct [23]. When these conflict [24], an appeal is to be made to common morality. Common morality is the organizing meta-principle and constraining principle of moral reasoning. A pertinent document of common morality is the Declaration of Helsinki’s principles of duty to the patient over society and the recognition of increased vulnerability of individuals or groups in research. While donor autonomy is to be respected as a standard, donor non-maleficence cannot be achieved with incentives because it may have no relation to regulation. Donor non-maleficence therefore trumps autonomy. Recipient beneficence is also trumped by donor non-maleficence because unlike with uncompensated donation, the donor has no interest in the recipient. Since financial gifts are untested in other surgical populations, donor non-maleficence also trumps beneficence to society.

The total moral burden of end-stage kidney disease belongs to the society that cannot provide enough donor kidneys. Acting with good reasons according to one arm of the dilemma, to increase kidney transplant numbers, does not erase obligations towards the other, to prevent donor harm. The second-order principle in this situation is that as moral agents, we must arrange our institutions to minimize such conflicts [25]. Increasing available donor kidneys from other sources is the only option. Promising initiatives in previously important incentive-based jurisdictions [3] to increase deceased donor transplantation [26] or more novel initiatives such as donation after cardiac death [27] and domino transplants [28] all act in accordance with this second-order principle. It requires considerable dedication and investment to act according to morally sound second-order principles. On the other hand, quickly creating a new group of at-risk incentivized donors only adds to the total moral burden of end-stage kidney disease.
